# YBa_2_Cu_3_O_y_ Superconducting Ceramics Incorporated with Different Types of Oxide Materials as Promising Radiation Shielding Materials: Investigation of The Structure, Morphology, and Ionizing Radiations Shielding Performances

**DOI:** 10.3390/nano12193490

**Published:** 2022-10-05

**Authors:** Essia Hannachi, Yassine Slimani, M. H. A. Mhareb, M. I. Sayyed, M. Kh. Hamad, Y. S. Alajerami, Nidal Dwaikat, Munirah A. Almessiere, Abdulhadi Baykal

**Affiliations:** 1 Department of Nuclear Medicine Research, Institute for Research and Medical Consultations (IRMC), Imam Abdulrahman Bin Faisal University, P.O. Box 1982, Dammam 31441, Saudi Arabia; 2 Department of Biophysics, Institute for Research and Medical Consultations (IRMC), Imam Abdulrahman Bin Faisal University, P.O. Box 1982, Dammam 31441, Saudi Arabia; 3 Department of Physics, College of Science, Imam Abdulrahman Bin Faisal University, P.O. Box 1982, Dammam 31441, Saudi Arabia; 4 Basic and Applied Scientific Research Center, Imam Abdulrahman Bin Faisal University, P.O. Box 1982, Dammam 31441, Saudi Arabia; 5 Department of Physics, Faculty of Science, Isra University, Amman 11622, Jordan; 6 Department of Basic Sciences, School of Social and Basics Sciences, Al Hussein Technical University, King Abdullah II St 242, Amman 11831, Jordan; 7 Medical Imaging Department, Applied Medical Sciences Faculty, Al Azhar University-Gaza, Gaza City P.O. Box 1277, Palestine; 8 Department of Physics, College of Engineering and Physics, King Fahd University of Petroleum & Minerals, Dhahran 31261, Saudi Arabia; 9 Interdisciplinary Research Center for Advanced Materials, King Fahd University of Peroleum & Minerals, Dhahran 31261, Saudi Arabia; 10 Department of Nanomedicine Research, Institute for Research and Medical Consultations (IRMC), Imam Abdulrahman Bin Faisal University, P.O. Box 1982, Dammam 31441, Saudi Arabia

**Keywords:** ceramic, XRD, morphology, shielding properties, proton, neutron

## Abstract

New series of YBCO ceramics samples doping with different oxides such as SiO_2_, WO_3_, Al_2_O_3_, and TiO_2_ were fabricated to study the ionizing radiation shielding properties. The structure and morphology were explored by X-ray diffraction (XRD) and scanning electron microscope (SEM). The shielding properties were investigated experimentally and theoretically to check the validity of the results. The investigated radiation shielding properties include the proton, neutron, and gamma-ray. The XRD results show the orthorhombic structure for all ceramics without any additional peaks related to WO_3_, SiO_2_, TiO_2_, and Al_2_O_3_. At the same time, the SEM results appear to have a significant differentiation in the granular behavior of all ceramics surfaces. The incorporation of WO_3_ to YBCO enhanced the ceramic density, whereas the addition of different oxides reduced the density for ceramic samples. This variation in density changed the radiation shielding results. The sample containing WO_3_ (YBCO-W) gives us better results in radiation shielding properties for gamma and neutron; the sample having Al_2_O_3_ (YBCO-Al) is superior in shielding results for charged particles. Finally, the possibility to use YBCO with various oxides in different ionizing radiation shielding fields can be concluded.

## 1. Introduction

Since discovering ionizing radiation (X-ray, gamma-ray, neutrons, and charged particles), many properties and applications of ionizing radiation are widely discussed in the literature [1,2,3,4,5,6,7,8]. These radiations have severe side effects and are hazardous to the environment and human’ and animals health. Workers in such a field need to apply the ALARA principle (as low as reasonably achievable) [9] and the three main radiation protection principles: increase the distance from the radiation sources, reduce the exposure time, and use the proper and effective radiation shielding materials. Conventionally, people use lead (Pb) and concrete-based materials for gamma- and x-ray shielding. However, Pb has many disadvantages, including toxicity, heavyweight, relatively nasty melting point, and low mechanical strength [10,11,12,13]. On the other hand, concrete can be easily cracked and affected by radiation exposure [14]. Therefore, it is important to search for other alternative materials to overcome these limitations.

In recent years, the synthesis of different radiation shielding materials, such as modified concrete [14,15,16], glasses [17,18], polymers [19], stainless steel [20], alloys [21,22], and ceramics [23,24,25], have begun to elicit increasing interest to enhance the radiation shielding properties in different applications. The goal is to achieve optimal values of various parameters, involving mass attenuation coefficient (MAC), half-value layers (HVL), and mean free path (MFP). Recently, ceramics have been extensively employed in medical applications due to their durability against high temperature, oxidation, and low thermal expansion [26]. Additionally, ceramics are eco-friendly, simple to make, inexpensive, and display low porosity. For example, SrMnO_3_ is a perovskite manganite that undergoes several metallic, insulating, and magnetic phases. It can be easily prepared and modified in the lab to be employed in different applications [24]. Erdem et al. [27] studied the radiation shielding properties of some perovskite ceramic materials. They conclude that the Cs_2_SnI_6_ composition is suitable as gamma-ray shielding material, while it is unsuitable for a neutron shielding field. In addition, Slimani et al. [24] examined the shielding characteristics for diverse ceramics; they improved the packing density value of SrMnO_3_ by adding tellurium. On another side, another group compared BaTiO_3_ and CaWO_4_ compounds and concluded these materials displayed an excellent capacity to stop gamma rays [28]. Another example, YBa_2_Cu_3_O_y_, is a ceramic with superconducting properties [29,30]. It can be used for several technological and power applications [31,32]. Adding various oxide materials to perovskite ceramics could enhance properties such as radiation shielding, superconducting performance, durability, structural and electrical properties, etc. In this work, some oxides such as tungsten oxide (WO_3_), silicon dioxide (SiO_2_), aluminum oxide (Al_2_O_3_), and titanium oxide (TiO_2_) were added to YBa_2_Cu_3_O_7-δ_. The choice of materials was based on the literature. WO_3_ owns a high atomic number, improving radiation shielding properties for any system, and it is considered a good choice instead of lead (PbO) [33]. The added Al_2_O_3_ and SiO_2_ drive an improvement in the mechanical and thermal stability of materials [34,35], while TiO_2_ can enhance the optical properties when added to different systems of ceramics. Furthermore, doping YBCO ceramic by appropriate metal oxides could lead to better dielectric properties, suggesting that such kind of ceramics could be employed for energy storage applications [36].

In the present work, we prepared a set of YBCO ceramic by the solid-state reaction to investigate their structural, morphological, and radiation shielding properties. To achieve this goal, we investigated the effects of adding different oxides (SiO_2,_ WO_3_, TiO_2_, and Al_2_O_3_) on the density and, hence, on both the structural and radiation properties at distinct applied energies. We used the Rietveld refinement method to analyze the X-ray diffraction (XRD) patterns. The morphology observations of the prepared ceramics were done using scanning electron microscopy (SEM), and the chemical analyses were done via the energy-dispersive X-ray spectroscopy (EDXS) system connected to the SEM. Additionally, the shielding features were determined experimentally and theoretically to check the validity of our results.

## 2. Experiment

The pure YBCO ceramic and ceramics with WO_3_, SiO_2_, TiO_2_, and Al_2_O_3_ additions were synthesized by the traditional solid-state reaction route in the same circumstances. All chemicals used in this study were purchased from Sigma Aldrich (Germany). The YBCO phase was made by meticulously mixing CuO (99.9%), BaCO_3_ (99.9%), and Y_2_O_3_ (99.9%) and conferring to the chemical formulation of Cu:Ba:Y = 3:2:1. The mix of these powders was compacted into pellets and afterward exposed to calcination at a temperature of 930 °C for 12 h to form a precursor of YBCO. The resulting pellets were hand-ground using an agate mortar to obtain precursor powder YBCO. In the second step, oxides of WO_3_, SiO_2_, TiO_2_, and Al_2_O_3_ were included in the precursor YBCO powder. The content of additives is 2.0 wt.% of the total mass of the ceramics. The four batches were compelled into 10-mm-diameter circular disks at 700 MPa. The obtained pellets were placed in alumina crucibles and next subjected to the sintering step at a temperature of 950 °C for 8 h. Finally, they cooled to room temperature at a rate of 3 °C/min. The pristine (i.e., 0%) powder was similarly ground as the added ceramics to safeguard the same physical circumstances for all the ceramics. Table 1 lists the labels, the phase fraction, and the chemical formula of each prepared ceramic. The density was measured based on Archimedes’ principle. The samples were weighed in air (*W_air_*) and then in liquid (*W_liq_*) and calculated by using the following relation:

(1) $$\rho =\frac{W_{air}}{W_{air}-W_{liq}}x\rho_{liq}$$

where $\rho_{liq}$ represents the liquid density. The distilled water was used as the immersion liquid.

The structure and the phase identification of the products were evaluated by a Benchtop Miniflex XRD (Rigaku, Tokyo, Japan). The diffraction data were registered in a diffraction angle range of 20–80°. The morphology of different ceramics was observed using a scanning electron microscopy (SEM) instrument (FEI Nova 200 NanoLab, Denton, TX, USA). The chemical analyses were done via energy-dispersive X-ray (EDX) spectroscopy system connected to the SEM.

The shielding features for current samples were studied experimentally to determine the linear and mass attenuation coefficient. Then the results were compared with XCOM results to check the samples’ validity and radiation shielding setup. Our previous research explained the experiment setup in detail [37], and the setup contains two gamma sources (^166^Ho and ^137^Cs) and scintillator detectors. The ^166^Ho gives us four energies (0.184, 0.28, 0.71, and 0.81 MeV), while ^137^Cs has one energy (0.662 MeV). The thallium-activated sodium iodide scintillator dimensions are 3 × 3, and this scintillator is linked to a pre-amplifier and amplifier. The transmitted and incident photons were collected using a multichannel analyzer with a resolution to reach 7% at 0.662 MeV.

After checking the samples and radiation shielding setup validation, the rest of the shielding properties was determined theoretically by XCOM [38], such as linear attenuation coefficient (LAC), mass attenuation coefficient (MAC), effective atomic number (Z_eff_), tenth value layer (TVL), and gamma transmission factor (TF). At the same time, the radiation shielding for charge particles was determined by determining the mass stopping power, the projectile range for electron and alpha particles by SRIM [39]. Lastly, the fast neutron removal cross-section was defined according to the Phy-X program [40].

## 3. Results and Discussion

### 3.1. Structural Investigation

**Figure 1:**
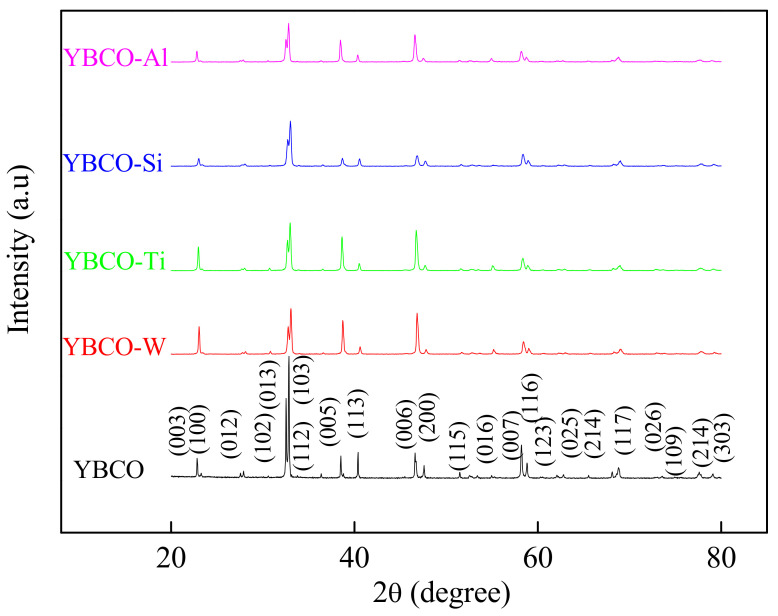
XRD patterns of YBCO, YBCO-W, YBCO-Ti, YBCO-Si, and YBCO-Al ceramics.

Figure 1 illustrates the powder XRD patterns of the ceramics of YBCO, YBCO-W, YBCO-Ti, YBCO-Si, and YBCO-Al. A mainly single YBCO phase with orthorhombic structure (Pmmm space group) is obtained. No peaks related to WO_3_, SiO_2_, TiO_2_, and Al_2_O_3_ or W, Si, Ti, and/or their derivatives were distinguished by XRD. In fact, lines with extremely minimal intensity, lower than five percent of the maximum intensity, come across in the patterns (sensitivity five percent) and combine into the noise backgrounds. Nevertheless, the EDX examination (see Figure 5b’–e’) indicates that W, Si, Ti, and Al elements exist in the ceramics. The lattice parameters for the prepared ceramics were determined.

**Figure 2:**
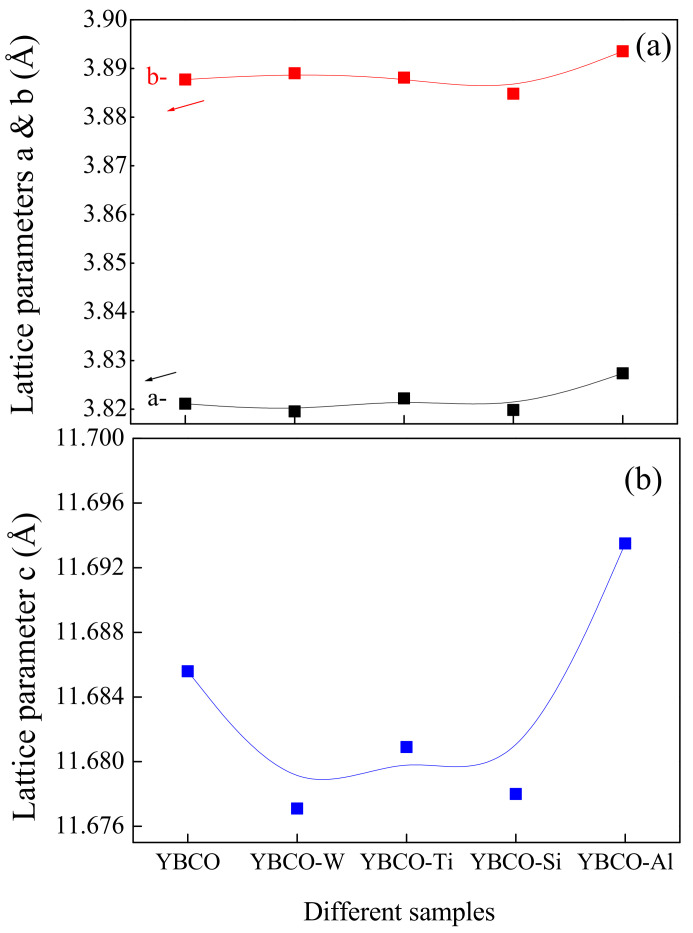
(**a**) Variations of the lattice parameters ‘a’ and ‘b’ for different ceramics. (**b**) Variations of the lattice parameter ‘c’ for different ceramics.

**Figure 3:**
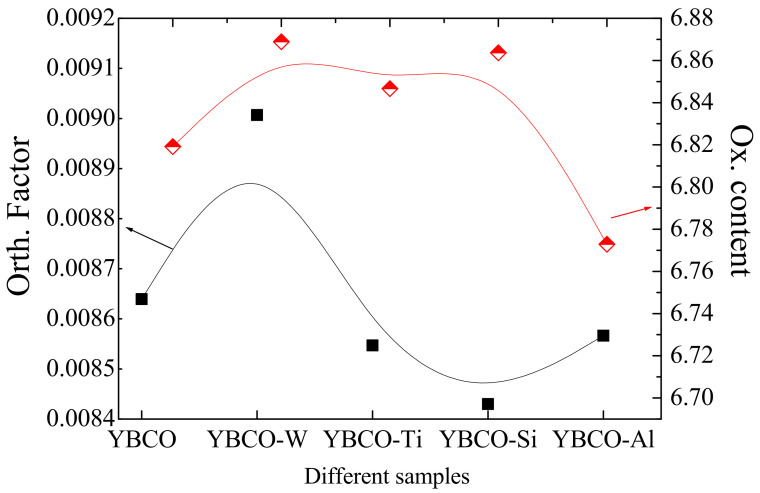
Variations of the Orth. factor (left axis) and O_x_ content (right axis) with different ceramics.

Figure 2 shows the evolution of the lattice parameters concerning different ceramic samples. The lattice parameters ‘a’ and ‘b’ show a similar tendency (Figure 2a). The lattice parameter ‘c’ shows a variation in the various ceramics (Figure 2b). Except for the YBCO-Al sample, the lattice parameters ‘a’ and ‘b’ are almost unchanged for YBCO-W, YBCO-Ti, and YBCO-Si. While the lattice parameter ‘c’ decreases for YBCO-W, YBCO-Ti, and YBCO-Si, it increases for YBCO-Al ceramics. There is no phase transition from orthorhombic to tetragonal detected under the XRD accuracy. However, the inclusion of SiO_2_, TiO_2_, and Al_2_O_3_ somewhat diminishes the difference between the lattice parameters a and b and, therefore, decreases the orthorhombic factor, i.e., (b − a)/(b + a), as revealed in Figure 3. The maximum orthorhombicity value is obtained in YBCO-W ceramics prepared with the WO_3_ addition. The value of the Ox. content acts as an adjusting factor of the structural and physical characteristics of the compound. The structure of YBCO progressively varies when the Ox. content varies: An Ox. content ranging between 6 and 6.5 values engenders the formation of the tetragonal phase. For Ox., a content value equal to 6.5 makes possible the transition from the tetragonal phase to the orthorhombic phase. Over an Ox. content equal to 6.8, a superconductive orthorhombic structure occurs [41]. Overall, among all the prepared ceramics, the YBCO-W sample prepared with a WO_3_ addition exhibits the highest Ox content and orth. The factor values reflect better properties in this ceramic. From the other side, the lattice parameter ‘c’ augmented and the Ox. content diminished in YBCO-Al ceramics containing Al_2_O_3_. The relationship between the lattice parameter ‘c’ and the Ox. content is in accordance with the common comportment of the lattice parameter ‘c’ for YBCO; that is to say, in the orthorhombic structure, the lattice parameter ‘c’ rises with the reduction of the Ox. content [41]. This result is consistent with those obtained by A. Mellekh et al. [42] in the case of YBa_2_Cu_3_Oy prepared by nano-Al_2_O_3_ particle addition.

**Figure 4:**
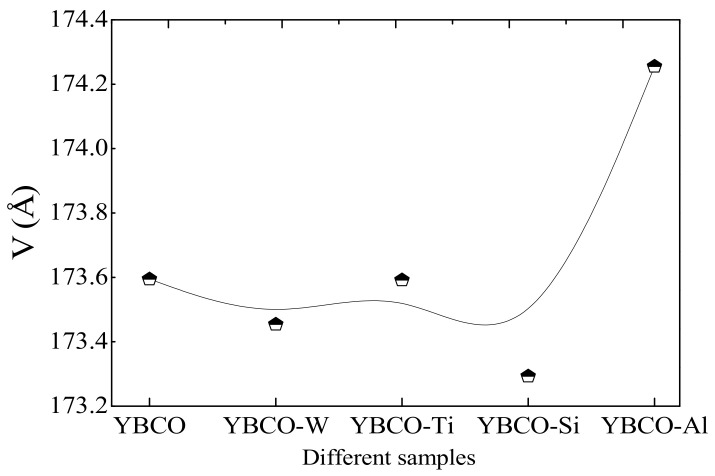
Variations of the lattice unit cell volume ‘V’ with different ceramics.

Furthermore, the unit cell volume ‘V’ changes in the different ceramics, as clearly shown in Figure 4. From this figure, the unit cell volume is greater for YBCO-Al than the other ceramics. Indeed, in the orthorhombic structure of the YBCO superconductor, the atoms of oxygen inside the basal planes are positioned at the O(1) sites and form Cu–O chains alongside the lattice parameter ‘b’. The O(5) sites persist unoccupied. The substitution of Cu^2+^ by Al^3+^ ions causes a reordering of oxygen atoms in the basal planes, as Al^3+^ did not sustain the square planar regularity. Consequently, an adjacent O ion will voyage from site O(1) to site O(5), which is vacant in the pristine YBCO [42]. For YBCO-Al containing Al_2_O_3_, the entire rise of relative ‘V’ is around 0.38%. This percentage is nearly the same as found by Siegrist et al. [43] in YBa_2_Cu_3_O_7_ ceramic samples prepared with Al substitution (relative V = 0.40%). Samples of YBCO-Si containing SiO_2_ and YBCO-W containing WO_3_ show a shrinkage in the unit cell volume ‘V’, which mostly could be due to the small ionic radii of Si ions and W ions in comparison to those of Cu ions. The unit cell volume ‘V’ slightly increases for YBCO-Ti containing TiO_2_ compared to pristine YBCO ceramics, which could be ascribed to the ionic radii of Ti ions that are slightly larger than those of the Cu ions.

### 3.2. Morphological Analysis

**Figure 5:**
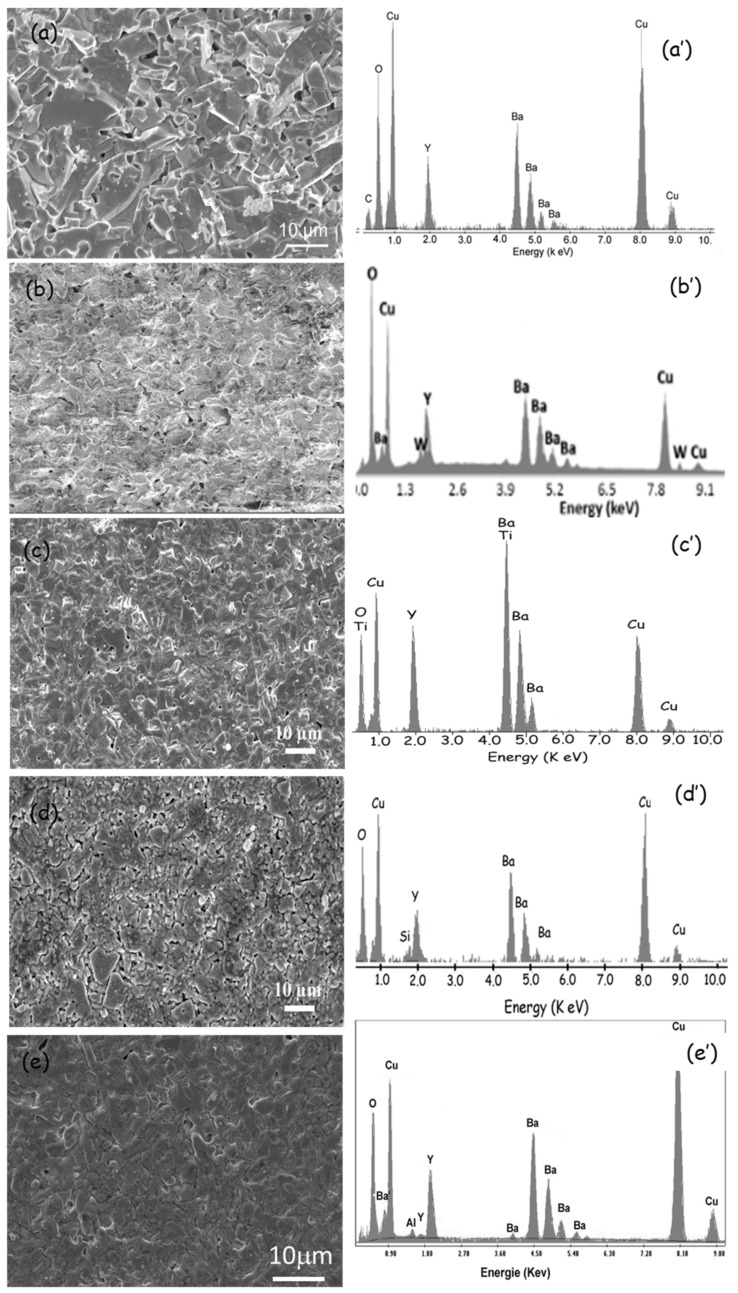
SEM images and EDX analysis for (**a,a’**) YBCO, (**b,b’**) YBCO-W, (**c,c’**) YBCO-Ti, (**d,d’**) YBCO-Si, and (**e,e’**) YBCO-Al.

In Figure 5, SEM images of pristine ceramic (Figure 5a) and WO_3_, SiO_2_, TiO_2_, and Al_2_O_3_ added ceramics (Figure 5b–e) are shown. According to the SEM results, a significant differentiation is observed in the granular behavior of the surfaces for all ceramics. For the YBCO ceramics (Figure 5a), the grain boundaries are quite clear, and the density of the pores is relatively high. Yet, for YBCO-W, YBCO-Ti, YBCO-Si, and YBCO-Al-added ceramics, the porosity is reduced. Figure 5a’–e’ show the EDX spectra of the prepared ceramics. EDX spectrum for pristine YBCO ceramics revealed the presence of Y, Ba, Cu, and O elements. The analyses for the added samples showed the presence of W, Si, Ti, and Al elements in addition to the elements detected for pristine YBCO ceramics. It can be concluded therefore from the SEM and EDX that the additions could alter the granular microstructure of the superconductor YBCO, which plays a crucial role in its functioning in potential applications, for example, radiation shielding purposes. This will be the objective of the next section.

### 3.3. Radiation Shielding Analysis

For the analysis of the radiation shielding of the fabricated ceramics, we measured the ability of these ceramics to attenuate the photons in the lab. This is a real prediction for the ceramic’s competence to block or attenuate the incoming photons, where we exposed the present ceramics to a beam of photons of different energies (0.184, 0.280, 0.662, 0.710, and 0.810 MeV), and with the help of the detector, we estimated the number of photons that were able to pass the ceramics (I_0_) and compared it to the total number of photons (without the presence of the ceramics; I). We then calculated the ratio between I and I_0_ and determined the experimental linear attenuation coefficient (LAC). This value was multiplied with density for the ceramic sample to get the MAC values. For the validation of the experimental part, we recalculated the MAC for these ceramics at the same energies used in the experiment. Still, this time, we did the calculations using XCOM software [44]. The MAC predicted from both approaches are enlisted in Table 2. The measured MAC matches the theoretical results; this is applicable for all ceramics at the examined energies. For instance, for YBCO, the measured MAC at 0.184 MeV is 0.4184 cm^2^/g, which is close to the XCOM result (0.4328 cm^2^/g). At higher energy and YBCO-Si, the MAC values are 0.06647 and 0.06926 cm^2^/g (at 0.81 MeV). Therefore, we can see the agreement in the measured and XCOM MAC at low and high energies. In the same table, we presented the deviation (RD) between the two mentioned methods for predicting the MAC for these ceramics. The RD varied between 0.2 and 5.09% at 0.184 MeV, between 1.14 and 5.81% at 0.280 MeV, between 1.73 and 6.08% at 0.662 MeV, between 2.08 and 5.64% at 0.710 MeV, and between 1.19 and 6.44% at 0.81 MeV. Therefore, the RD is less than 7%, which reaffirms that the setup used in the lab to predict the MAC for the YBCO ceramics and WO_3_, SiO_2_, TiO_2_, and Al_2_O_3_-added ceramics gives accurate results and is consistent with the theoretical data.

**Figure 6:**
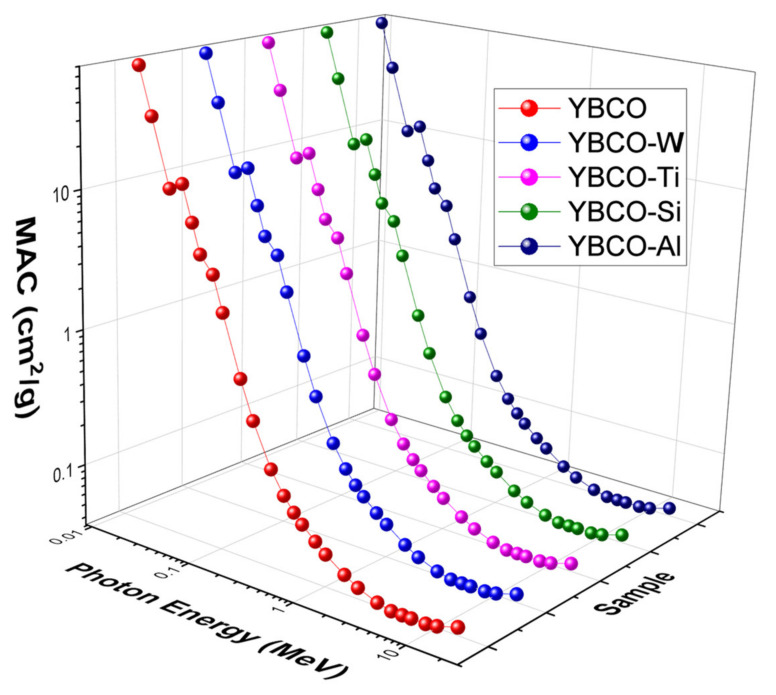
MAC values for the produced ceramics.

Experimentally, we determined the MAC at energies between 0.184 and 0.81 MeV since these energies are emitted from ^137^Cs and ^166^Ho sources, and these are available in our lab. However, it is essential to investigate the radiation shielding properties for these ceramics at other energies (lower and higher energies than the used energy range) and to understand the shielding performances of these ceramics at lower and higher energies. Therefore, we again used XCOM to extend the energy range and determine the MAC between 0.015 and 15 MeV. We plotted the expanded results in Figure 6. The MAC of the five prepared ceramics has a similar profile, since they have almost the same composition. At 15 keV, the MAC is high and takes the values: 69.83 cm^2^/g for YBCO and 70.08, 69.72, 69.72, and 69.65 cm^2^/g for WO_3_, SiO_2_, TiO_2_, and Al_2_O_3_-added ceramics. These values quickly dropped to 32.45, 32.57, 32.40, 32.40, and 32.36 cm^2^/g for the mentioned ceramics. This quick drop in the MAC is accepted due to the predominant photoelectric effect. As a result, the attenuation competence of the five ceramics is high at the first few energies. While this competence to shield the photons is reduced for the moderate energy, as well as high energy.

**Figure 7:**
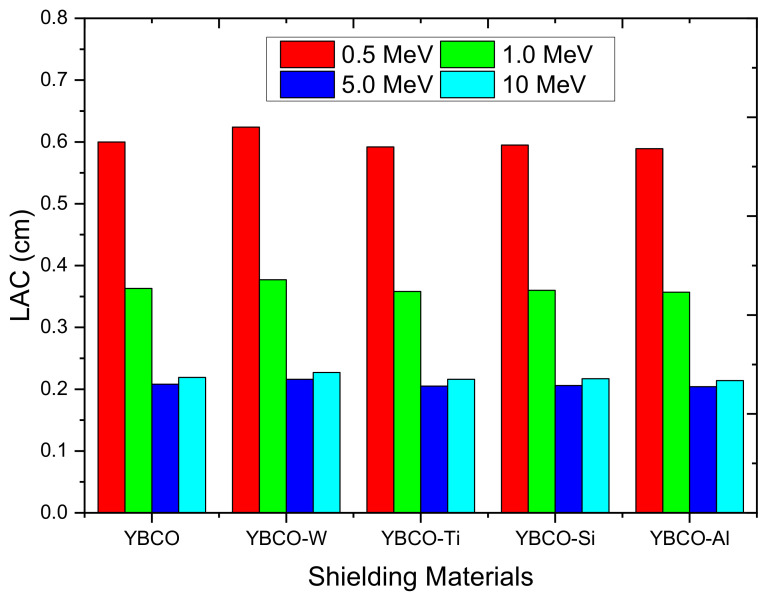
LAC values for the produced ceramics at 0.5, 1, 5, and 10 MeV.

In Figure 7, we presented the linear attenuation coefficient (LAC) results at four energies (i.e., 0.5, 1, 5, and 10 MeV). The first observation from this figure totally agrees with the MAC curves; the LAC has high values at 0.5 MeV and then decreases for the two energies 1 and 5 MeV. For the last energy (10 MeV), we found the LAC slightly increased, which is ascribed to the predominant pair production. The second observation is the influence of WO_3_, SiO_2_, TiO_2_, and Al_2_O_3_ on the LAC values. Apparently, if we compare the LAC for the YBCO to that of the WO_3_, SiO_2_, TiO_2_, Al_2_O_3_-added ceramics, we can see that only the ceramic with WO_3_ has a higher LAC than the YBCO. For example, the LAC values for YBCO and YBCO-W are 0.600 and 0.624 cm^−1^ (at 0.5 MeV), 0.363 and 0.377 cm^−1^ (at 1 MeV), 0.208 and 0.216 cm^−1^ (at 5 MeV), and 0.219 and 0.227 cm^−1^ (at 10 MeV). The addition of WO_3_ to the YBCO provokes an enhancement in the LAC and, hence, on the attenuation competence for the ceramic. This finding recommends incorporating other heavy metal oxides into the YBCO to get ceramics with interesting radiation shielding properties.

**Figure 8:**
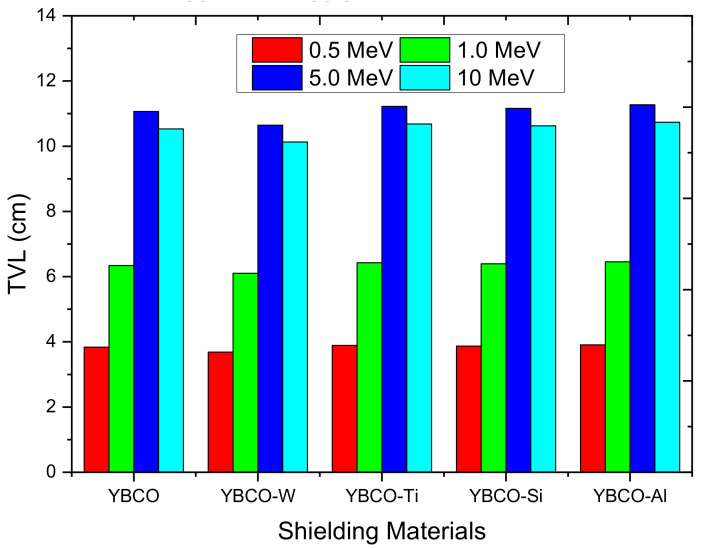
The tenth value layer for the produced ceramics at 0.5, 1, 5, and 10 MeV.

In addition to the MAC and LAC, it is helpful to estimate the thickness of the ceramic that can effectively attenuate the radiation to some level. Some penetrating parameters such as the tenth value layer (TVL) can be considered for this aim. TVL is one of the most frequently utilized quantities for describing the penetrating ability of particular photons, as well as the penetration through a specific medium. The TVL for the YBCO and WO_3_, SiO_2_, TiO_2_, and Al_2_O_3_-added ceramics at the energies used in the previous figure is graphed in Figure 8. The minimum TVL as given in this figure is found for the photon with an energy of 0.5 MeV. This is the smallest energy selected in Figure 8, and about 4 cm of the prepared ceramics is required to shield most of the incoming radiation. When the energy increases to 1 MeV, the thickness of the ceramics becomes about 6 cm. Increasing the thickness generally increases the probability of interactions (attenuation) and, hence, decreases the penetration.

In conclusion, a thick ceramic sample is an excellent absorber to the photons, and thus, the photons are less penetrating to this thick sample. This is correct for the low and moderate energy regions, while in the pair production region, there are limits and exceptions to this observation. For this reason, we found that the TVL at 10 MeV is less than that of 5 MeV.

**Figure 9:**
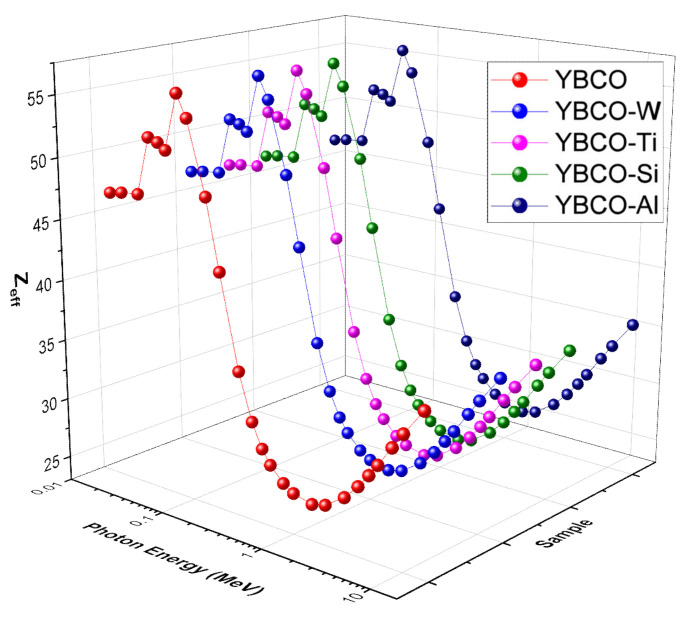
Z_eff_ values for the prepared ceramics.

We represented the Z_eff_ values for pristine ceramic and WO_3_, SiO_2_, TiO_2_, and Al_2_O_3_-added ceramics in Figure 9. The trend in Z_eff_ could be divided into three parts. Each region occurs due to the domination of photon interaction phenomenon [45]. At low energies, the photoelectric effect is the most dominant phenomenon. In this energy region, Z_eff_ increases slowly between 0.015 and 0.1 MeV and then drastically decreases. All ceramics contain Yttrium (Y, Z = 39, K-absorption edge at 0.017 MeV, between the first and second selected energies), so the Z_eff_ starts at high values, and due to the presence of Ba (which has a K absorption edge at 0.0374 MeV), the Z_eff_ increases slightly increases near this energy. As the energy increases beyond 0.08 MeV, the Z_eff_ diminishes up to 2 MeV. In the second region, the photoelectric effect becomes less dominant, and Compton scattering overtakes it. Z_eff_ is only slightly inversely proportional to the energy in this medium energy range, which explains the more gradual decrease in values. The Z_eff_ values continue to drop until 3 MeV, where the pair production process is being the dominant photon interaction phenomenon. Z_eff_ is directly proportional to the energy in this energy range, which accounts for the slight increase in values between 3 and 15 MeV. We selected YBCO (as an example) and summarized the previous trend using some numerical values. The Z_eff_ for YBCO equals 46.84 at 0.015 MeV and increases to 47.22 at 0.03 MeV and 55.79 at 0.08 MeV. The Z_eff_ values continue decreasing to 42.48 at 0.2 MeV, 28.04 at 0.6 MeV, 26.35 at 1 MeV, and 26.27 at 2 MeV. Between 3 and 15 MeV, the Z_eff_ increases from 27.39 to 36.01. Since Z_eff_ generally describes the shielding capability of a material, based on these values, the glasses have a much greater attenuation potential at low energies. In all three energy ranges, however, YBCO-W has the greatest Z_eff_, because it has WO_3_, and the atomic number of W is high.

**Figure 10:**
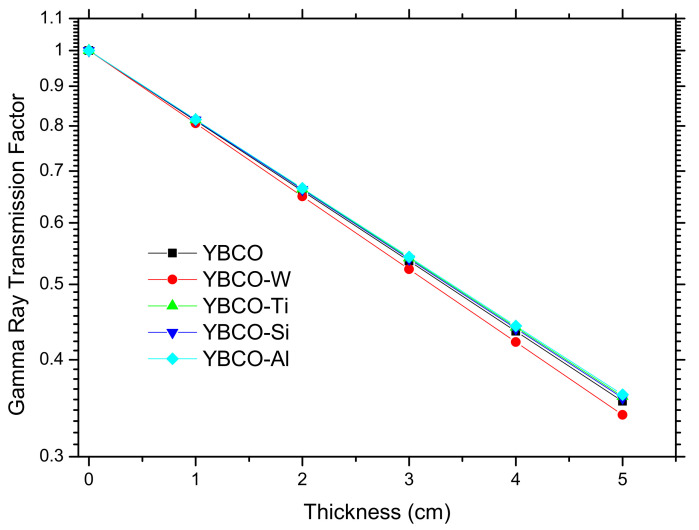
Transmission factor for the prepared ceramics versus the thickness.

The transmission factor (TF) for the prepared ceramics is also investigated, since it represents an excellent tool to examine the attenuation competence of these ceramics. The TF of the ceramic is the ratio of the intensity of the radiation passing across the ceramic to the intensity (I_0_) incident upon the ceramic surface: TF = I/I_0_. In Figure 10, we plotted the results of the TF for the pristine ceramic and WO_3_, SiO_2_, TiO_2_, and Al_2_O_3_-added ceramics. It is evident the inverse relationship between the number of photons that can pass through the ceramic and its thickness. For a small thickness, most of the photons can easily pass through all the ceramics. Thus, the attenuation competence is weak. Increasing the thickness enhances the attenuation competence (the penetration is decreased, as the penetration is the inverse of attenuation). At a thickness of 5 cm, the TF is in the order of 33% (this means that more than 66% of the photons cannot pass through the samples). Additionally, the TF figure shows that the YBCO-W has better attenuation performance than the other ceramics due to the lower TF.

**Figure 11:**
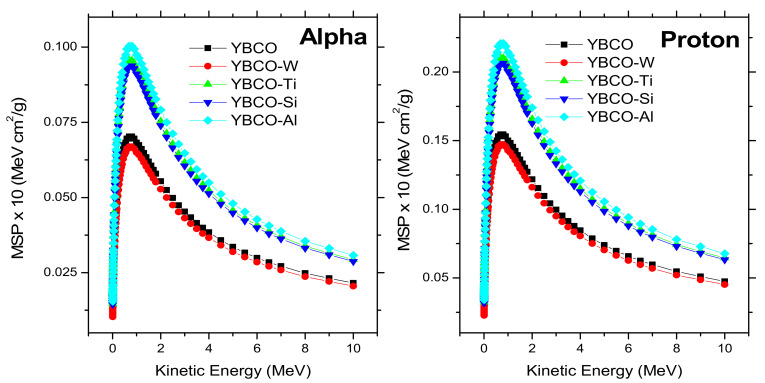
MSP values for proton and alpha versus the kinetic energy for fabricated ceramics.

**Figure 12:**
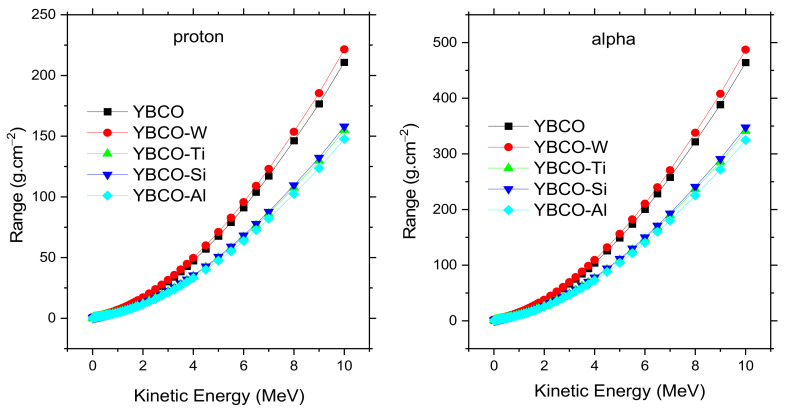
The range for proton and alpha versus the kinetic energy for fabricated ceramics.

Figure 11 displays the mass stopping power (MSP) with changes in the kinetic energy for differently charged particles: alpha and protons. From Figure 11, the similarities in the shapes of MSP for alpha and protons particles can be noted, but the values of MSP for alpha particles are lower than the values of MSP for protons. For example, the MSP values for alpha and protons for YBCO at 0.7 MeV are 0.7025 and 1.5456 MeV cm^2^/g, respectively. On the other hand, the effect of density values on MSP is clear in Figure 11. The MSP at the maximum peak (0.7 MeV) for protons is 1.5456, 1.472, 2.1029, 2.0636, and 2.208 MeV cm^2^/g for YBCO, YBCO-W, YBCO-Ti, YBCO-Si, and YBCO-Al. From Table 1, we can note that the YBCO-Al has the lowest density. In contrast, YBCO-Al has the highest MSP, and all samples follow this trend. This trend can be explained that the heavy atoms that contributed to the fabrication of different samples have electrons tightly bound to their orbits, leading to a reduction of MSP values for heavy composite materials. Figure 12 illustrates the range (R) for alphas and protons, and the range is considered the reciprocal of the MSP and multiplied by energy. The obtained results show the highest R for YBCO-W compared to other samples for both charged particles. This result is compatible with the MSP results. According to the MSP results, the heavy composite materials have low MSP values, because a strong bond exists between the electrons and their orbitals. Based on this analysis, the heavy composite has less absorption for charged particles.

**Figure 13:**
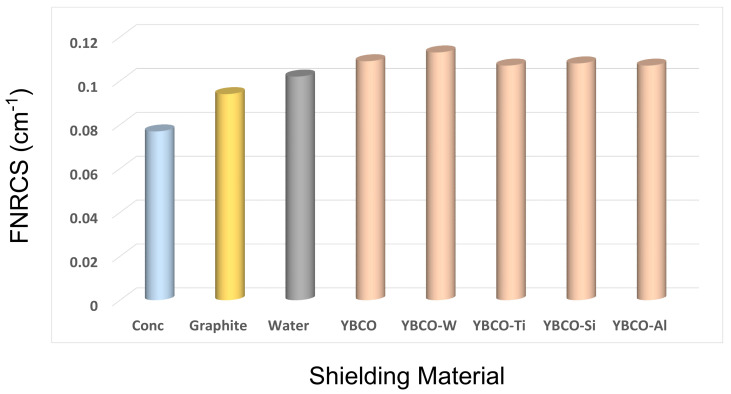
The comparison between the FNRCS values for fabricated samples and standard materials.

Figure 13 shows the fast neutron removal cross-section (FNRCS) comparison for the current samples and some of the standard materials. According to the obtained results, the YBCO-W sample has the highest FNRCS value in comparison to other ceramics, and this sample is superior to other samples in stopping fast neutrons. The values of FNRCS are 0.113, 0.109, 0.108, 0.107, 0.107, 0.102, 0.094, and 0.077 cm^−1^ for YBCO-W, YBCO, YBCO-Si, YBCO-Ti, YBCO-Si, water, graphite, and concrete, respectively. Here, it can be noted that the FNRCS for fabricated samples is compatible with the gamma shielding results and inverse for the charged particle shielding results.

## 4. Conclusions

In this study, we performed a comparative study on the effects of doping of different oxides on the structure, morphology, and radiation shielding properties of a YBCO superconductor. Five samples of YBCO added with diverse oxides were fabricated by the solid-state reaction. The XRD results did not show any change in the peaks with the addition of different oxides, and all samples displayed an orthorhombic structure and Pmmm space group. A significant change in granular behavior for surface for all ceramics samples was observed. The addition of WO_3_ to YBCO enhanced the density and improved the gamma and neutron shielding properties. The MAC properties were determined experimentally and compared with XCOM data, and the maximum relative difference (RD) between the experimental and XCOM data is 6.44%. This value is located within the acceptable results. The gamma shielding properties for the YBCO-W sample are highest compared with other samples. For example, the MAC values at 15 keV are 70.08, 69.83, 69.72, 69.72, and 69.65 cm^2^/g for YBCO-W, YBCO, YBCO-Si, YBCO-Ti, and YBCO-Al. The FNRCS results showed superior results for YBCO-W compared to the other samples, and the results of FNRCS were as follows: 0.113, 0.109, 0.108, 0.107, 0.107, 0.102, 0.094, and 0.077 cm^−1^ for YBCO-W, YBCO, YBCO-Si, YBCO-Ti, YBCO-Si, water, graphite, and concrete, respectively. At the same time, the MSP at 0.7 MeV for protons is 2.208, 2.1029, 2.0636, 1.5456, and 1.472 MeV cm^2^/g for YBCO-Al, YBCO-Ti, YBCO-Si, YBCO, and YBCO-W. From the obtained results, we can nominate YBCO-W to be a radiation shielding material for neutrons and gamma rays and YBCO-Al to be a radiation shielding material for protons.

## Figures and Tables

**Table 1:** The labels, the phase portion, and the chemical formula of each prepared ceramic.

Sample Code	Formula	Phase Fraction (%)		Density g/cm^3^
**YBCO**	YBa_2_Cu_3_O_7-δ_	100% YBa_2_Cu_3_O_7-δ_	-	6.0021
**YBCO-W**	(YBa_2_Cu_3_O_7-δ_)/(WO_3_)_2_	98% YBa_2_Cu_3_O_7-δ_	2% WO_3_	6.2345
**YBCO-Ti**	(YBa_2_Cu_3_O_7-δ_)/(TiO_2_)_2_	98% YBa_2_Cu_3_O_7-δ_	2% TiO_2_	5.9213
**YBCO-Si**	(YBa_2_Cu_3_O_7-δ_)/(SiO_2_)_2_	98% YBa_2_Cu_3_O_7-δ_	2% SiO_2_	5.9521
**YBCO-Al**	(YBa_2_Cu_3_O_7-δ_)/(Al_2_O_3_)_2_	98% YBa_2_Cu_3_O_7-δ_	2% Al_2_O_3_	5.8945

**Table 2:** Comparison among theoretical and experimental values of MAC at diverse energies.

Energy (MeV)		YBCO	YBCO-W	YBCO-Ti	YBCO-Si	YBCO-Al
**0.184**	**EXP**	0.4184	0.4209	0.4541	0.4158	0.4311
	**XCOM**	0.4328	0.4319	0.4321	0.4323	0.4350
	**RD%**	3.32	3.24	5.09	3.81	0.20
**0.280**	**EXP**	0.1973	0.1888	0.1970	0.2039	0.2018
	**XCOM**	0.1998	0.1995	0.1996	0.1996	0.2005
	**RD%**	1.25	5.81	1.32	2.14	1.14
**0.662**	**EXP**	0.07832	0.08312	0.07685	0.07485	0.08420
	**XCOM**	0.07969	0.07968	0.07968	0.07969	0.07978
	**RD%**	1.73	4.18	3.55	6.08	5.68
**0.710**	**EXP**	0.07150	0.07427	0.07209	0.07400	0.07818
	**XCOM**	0.07577	0.07577	0.07576	0.07577	0.07585
	**RD%**	5.64	2.08	4.84	2.33	3.18
**0.810**	**EXP**	0.07007	0.07371	0.07069	0.06647	0.06547
	**XCOM**	0.06925	0.06925	0.06925	0.06926	0.06931
	**RD%**	1.19	6.44	2.08	4.03	5.54

## Data Availability

Not applicable.
